# Neoadjuvant chemotherapy and Avelumab in early stage resectable nonsmall cell lung cancer

**DOI:** 10.1002/cam4.3456

**Published:** 2020-09-29

**Authors:** Arafat Tfayli, Majd Al Assaad, Ghina Fakhri, Reem Akel, Hanine Atwi, Hady Ghanem, Fadi El Karak, Fadi Farhat, Kamal Al Rabi, Pierre Sfeir, Pierre Youssef, Ziad Mansour, Hazem Assi, Mohamad Haidar, Alain Abi Ghanem, Ibrahim Khalifeh, Fouad Boulos, Ramy Mahfouz, Bassem Youssef, Youssef Zeidan, Rachelle Bejjany, Fadlo Khuri

**Affiliations:** ^1^ Division of Hematology‐Oncology American University of Beirut Medical Center Beirut Lebanon; ^2^ Department of Internal Medicine Lebanese American University Medical Center‐Rizk Hospital Beirut Lebanon; ^3^ Department of Internal Medicine Saint Joseph University Beirut Lebanon; ^4^ Division of Hematology‐Oncology Hammoud Hospital University Medical Center Saida Lebanon; ^5^ Department of Internal Medicine King Hussien Cancer Center Amman Jordan; ^6^ Division of Cardiothoracic Surgery American University of Beirut Medical Center Beirut Lebanon; ^7^ Division of Cardiothoracic Surgery Hammoud Hospital University Medical Center Saida Lebanon; ^8^ Division of Cardiothoracic Surgery Geitaoui Medical Center Beirut Lebanon; ^9^ Department of Radiology American University of Beirut Medical Center Beirut Lebanon; ^10^ Department of Pathology American University of Beirut Medical Center Beirut Lebanon; ^11^ Department of Radiation Oncology American University of Beirut Medical Center Beirut Lebanon

**Keywords:** immune checkpoint inhibitors, neoadjuvant therapy, nonsmall cell lung cancer, oncogenic drivers

## Abstract

Multiple randomized studies have shown that combination of chemotherapy and immune checkpoint inhibitors (ICIs) leads to better response rates and survival as compared to chemotherapy alone in the advanced stage of NSCLC. Data suggesting a benefit to using ICIs in the neoadjuvant therapy of patients with early stage NSCLC are emerging. Eligible subjects were treatment naïve patients with stage IB, II, and resectable IIIA NSCLC. Patients received three cycles of neoadjuvant chemotherapy with four doses of avelumab every 2 weeks. Patients with squamous cell cancer received cisplatin or carboplatin on day 1 and gemcitabine on days 1 and 8 of each cycle of chemotherapy. Patients with nonsquamous histology received cisplatin or carboplatin with pemetrexed on day 1 of each cycle. Patients then proceeded to their planned surgery. Out of 15 patients accrued as part of stage 1 of the study, four had a radiologic response (1 complete response), lower than the minimum of six responses needed to continue to phase 2 of the study. The study was therefore terminated. Majority had adenocarcinoma histology and stage IIIA disease. The treatment was well tolerated with no unexpected side effects. Four patients (26.7%) had grade III/IV CTCAE toxicity. This study confirms that the preoperative administration of chemotherapy and avelumab is safe. There was no indication of increased surgical complications. The benefit of adding immunotherapy to chemotherapy did not appear to enhance the overall response rate of patients in the neoadjuvant setting in patients with resectable NSCLC because this study failed to meet its primary endpoint.

## Graphical abstract

In this clinical trial, patients with resectable early stage non‐small cell lung cancer received a combination of neoadjuvant chemotherapy with avelumab, an anti‐PD‐L1 monoclonal antibody. The study found that there were no increased surgical complications. Also, the benefit of adding immunotherapy to chemotherapy did not appear to enhance the overall response rate of patients in the neoadjuvant setting in patients with resectable NSCLC because this study failed to meet its primary endpoint.

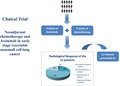

## 1 INTRODUCTION

Lung cancer is the leading cause of cancer death worldwide with nonsmall cell lung cancer (NSCLC) comprising approximately 85% of cases. The introduction of immune checkpoint inhibitors (ICIs) have consistently led to better outcomes in patients with stage IV and IIIB disease.^1^, ^2^, ^3^, ^4^, ^5^, ^6^, ^7^ More recently, multiple randomized studies have shown that combination of chemotherapy and immunotherapy leads to better response rates and survival as compared to chemotherapy alone in advanced stage NSCLC.^8^, ^9^, ^10^

Patients who present with early stage, potentially resectable, disease have better outcomes. However, a significant proportion of these patients still develop recurrent disease and succumb to their illness. Adjuvant therapy has been shown to modestly improve outcomes in this patient population.^11^, ^12^, ^13^, ^14^, ^15^ Neoadjuvant therapy has the advantage of early treatment of micrometastatic disease and in allowing for a radiologic and pathologic evaluation of response to therapy. Data suggesting a benefit to the use of ICIs in the neoadjuvant therapy of patients with early stage NSCLC are starting to emerge.^16^ We hypothesize that combining neoadjuvant chemotherapy with immunotherapy may provide additional benefits; beyond the simple additive benefit of two effective therapies, tumor cell killing by chemotherapy may increase tumor accessibility to the immune system and may increase tumor antigen shedding leading to a more effective antitumor immune response. We report the initial data of an open‐label multicenter study using combination of platinum‐based doublet chemotherapy in combination with Avelumab (Merck KGa) as neoadjuvant therapy in patients with early stage NSCLC. Avelumab is currently not FDA‐approved for treatment of NSCLC. This study is a clinical trial registered in ClinicalTrials.gov ID under the ID “**NCT03480230”.**

## 2 PATIENTS AND METHODS

### 2.1 Patients

Eligible subjects were treatment naïve patients with stage IB (>4 cm in size), II, and resectable IIIA NSCLC as determined by a whole‐body PET scan done for each subject at baseline according to the TNM staging for lung cancer, 8th edition. Subjects had to be 18 years or older with an Eastern Cooperative Oncology Group (ECOG) performance status of 0 or 1.

The subjects must have an adequate cardiac, pulmonary, renal, hepatic, and hematologic function. Prior to cardiac and pulmonary clearance for thoracic surgery, extent of surgery (pneumonectomy, lobectomy, wedge resection) was determined by a thoracic surgeon at baseline. Cardiac function was assessed adequately at baseline by a specialist and every patient with a significant active cardiac disease was excluded at screening. Pulmonary function was also assessed by a specialist at baseline with adequate pulmonary function tests. Patients were enrolled irrespective of tumor PD‐L1 status. Patients were excluded if they had history of auto immune diseases necessitating systemic therapy, history of hepatitis B, C or HIV. Patient who received prior ICIs were also excluded. The study obtained institutional board review/ethics committee approval at each study site. All patients signed written informed consents. The study is investigator initiated.

### 2.2 Study design and treatment

Patients received three cycles of neoadjuvant chemotherapy with four doses of avelumab at 10 mg/kg every 2 weeks starting on day 1 of chemotherapy. Patients with squamous cell cancer received cisplatin at 75 mg/m^2^ or carboplatin (AUC 5) on day 1 and gemcitabine at 1000 mg/m^2^ on days 1 and 8 of each cycle of chemotherapy. Patients with nonsquamous histology received cisplatin at 75 mg/m^2^ or carboplatin (AUC 5) on day 1 with pemetrexed at 500 mg/m^2^ on day 1 of each cycle. Patients were reassessed posttreatment with a chest CT scan. The response to treatment was determined by comparison of this CT scan with the baseline imaging according to Response Evaluation Criteria in Solid Tumors (RECIST) version 1.1. Patients were re‐evaluated by the thoracic surgeon posttreatment to determine the extent of thoracic surgery (pneumonectomy, lobectomy, wedge resection). Patients were also assessed posttreatment by pulmonary medicine and cardiology specialist to determine their eligibility for surgery. Patients who were cleared for the required thoracic surgery proceeded to it, patients who were not cleared, continued to be treated as per standard of care. No adjuvant chemotherapy or immunotherapy was given. Patients with mediastinal lymph node involvement received postoperative mediastinal radiation as per standard of care.

### 2.3 Study endpoints

The primary endpoint of the study was overall response rate (ORR) using the RECIST version 1.1 criteria. According to the imaging results, patients were divided into four groups as follows: responsive disease, stable disease, locally progressive disease, and locally advanced or metastatic progressive disease. Secondary endpoints included complete pathologic response rate, major pathologic response rate (<10% viable tumor cells), and progression‐free and overall survival. Preplanned analyses included assessing ORR, PFS, and OS according to histology and PD‐L1 status (22C3 antibody), patient‐related outcomes, and tolerability.

### 2.4 Statistical analysis

With an estimated response rate of 55% (p1 = 55%), a width of ±14% for the 95% confidence interval for the response rate, and a drop‐out rate of 20%, the sample size needed was 60 patients. This sample size would allow for adopting a Simon's two‐stage design which permits early termination of the study in case the study treatment was not effective. Stage 1 would include enrolling 15 patients. If six or fewer patients responded in stage 1, the study would be terminated. If seven or more patients have a response (>40% response rate, p0 = 40%), we planned to proceed with stage 2 up to 60 patients in total.

Except for the analysis of the two‐stage Simon's design that uses a one‐sided significance level of 0.05, a two‐sided *P*‐value of .05 was used for statistical significance, and all statistical analyses will be carried out using SAS. Then, the ORR of patients receiving neoadjuvant avelumab plus platinum doublet chemotherapy would be calculated along with its 95% confidence interval.

Secondary analysis included similar analyses for the pathologic complete response and estimation of the median PFS along with 1‐, 2‐, and 3‐year PFS and median OS using the Kaplan‐Meier methods.

Frequency distribution for AEs and SAEs was assessed per cycle and per patient.

## 3 RESULTS

**FIGURE 1:**
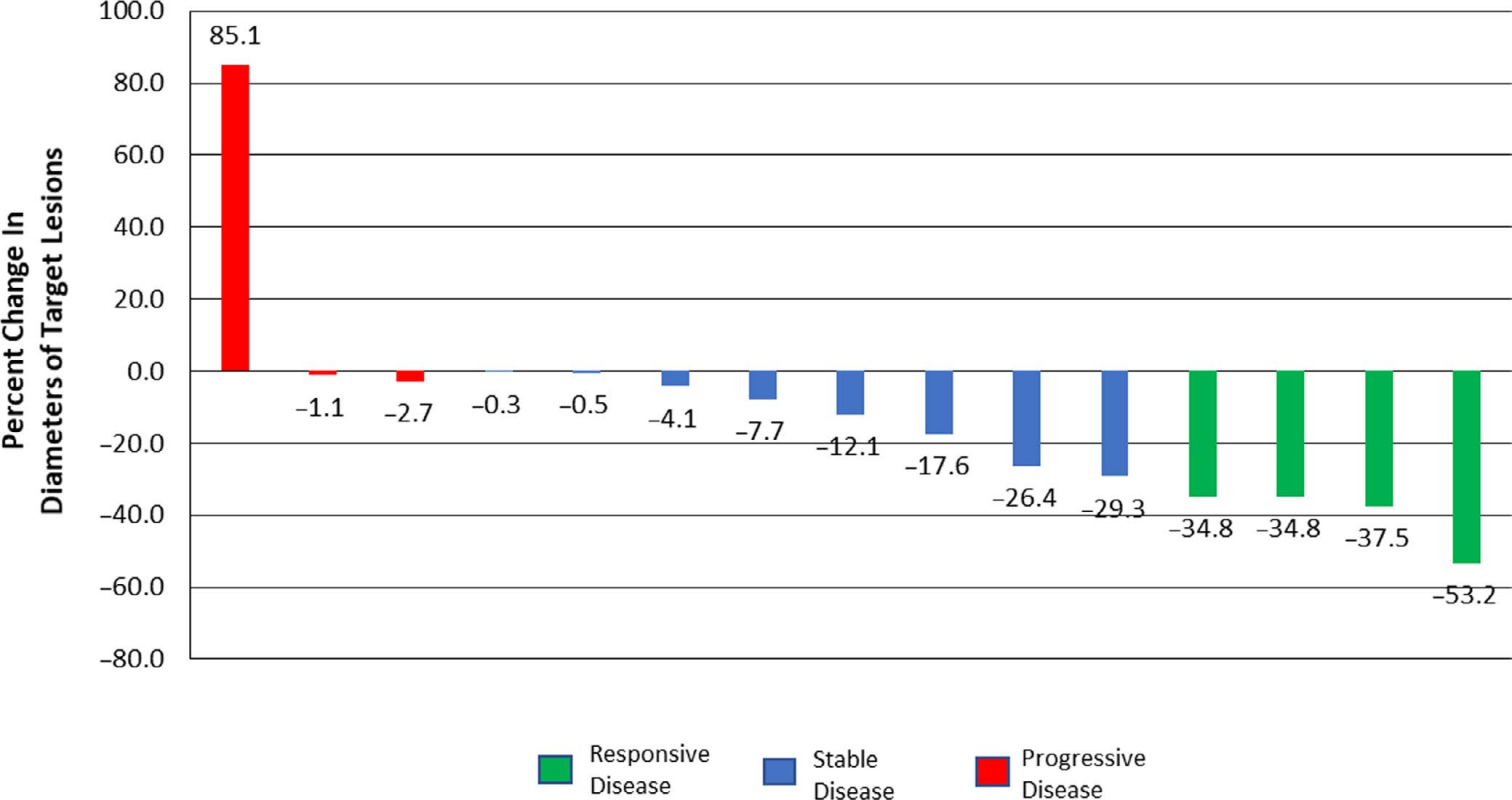
Percent Change in Diameters of Target Lesions

As per the statistical design, 15 patients were accrued at five sites as part of stage one of the study between July 2018 and April 2019. As of data cutoff—December 10, 2019—the median duration of follow‐up is 10 months. The median treatment duration is 9 weeks. Out of the 15 patients, four had a radiologic response, one complete response (CR) and three partial responses (PR) (Figure 1), which is lower than the minimum of six responses needed to continue to phase 2 of the study. The study was therefore terminated. Out of the 15 patients, 11 have done thoracic surgery therefore, their resected tumor could be assessed for pathological response. Out of those 11 patients, two patients had a partial pathological response and one patient had a complete pathological response (Figure 1, Table 2). As shown in Table 1, the patients’ median age was 65 years with almost equal distribution among males and females. Majority of patients were current or former smokers (73%), had an ECOG performance score of 0 (80%), adenocarcinoma histology (86.7%), and stage IIIA disease (53.3%).

**TABLE 1:** Patient demographics and disease characteristics

Variable	N = 15 (%)
Median age, years (range)	65 (45‐80)
Gender	
Male	7 (46.7)
Female	8 (53.3)
ECOG PS	
0	12 (80)
1	3(20)
Smoking history	
Current or former	11 (73.3)
Never	4 (26.7)
Histology	
Squamous	2 (13.3)
Adenocarcinoma	13 (86.7)
Disease stage	
High risk IB	2 (13.3%)
II	5 (33.3%)
III	8 (53.3%)

Median progression‐free survival and median overall survival are not reached yet.

### 3.1 Safety

The treatment was well tolerated with no unexpected side effects. Four patients (26.7%) had grade III/IV CTCAE toxicity. These included neutropenia and thrombocytopenia (n = 1), headache and abdominal pain (n = 1), seizure (n = 1), pulmonary embolism (n = 1). No increased surgical complications including wound infections or dehiscence were noticed. Four patients did not undergo resection (one had progressive disease, three needed a pneumonectomy, and their PFTs were not good enough to do that). No patients experienced autoimmune complications including pneumonitis.

## 4 DISCUSSION

To our knowledge, this is the first study reporting on the efficacy of combination of chemotherapy and immunotherapy in patients with early stage, resectable NSCLC. This clinical trial failed to achieve the primary endpoint and it failed to prove the efficacy of adding avelumab immunotherapy to platinum‐based chemotherapy in the neoadjuvant treatment of patients with nonsmall cell lung cancer irrespective to the tumor PD‐L1 expression. Despite the failure to achieve the response rate needed to continue the study, multiple observations were noticed with detailed analysis of the data. Table 2 lists the individual patients according to stage, tumor histology, and tumor PD‐L1 expression. Of note, 13 out of the 15 patients had adenocarcinoma with only two having a PR, while both patients with squamous cell histology responded including one CR. The patient numbers are too small to make firm conclusions from this, but the association is thought provoking and warrants looking into this pattern in larger studies. Second, 9 out of 13 patients who had enough tumor cells for PD‐L1 testing had PD‐L1‐negative tumors. Out of these, only one responded to neoadjuvant chemotherapy and avelumab. In contrast, three out of the four patients with PD‐L1‐positive tumors responded. Also, three out of the 15 patients were treated with cisplatin and 12 with carboplatin as a choice of platinum doublet with the immunotherapy. Out of the three patients who received cisplatin as part of the treatment, one patient had a PR and two patients had stable disease (SD) as per RECIST 1.1. Out of the 12 patients who received carboplatin, three patients had PR and six patients had SD. The number of patients is too small to reveal a superiority of cisplatin or carboplatin when combined with immunotherapy in the neoadjuvant setting patients with resectable NSCLC.

**TABLE 2:** Individual patient tumor characteristics and response

Patient number	Clinical stage	Histopathology	Platinum Doublet	PD‐L1 expression	Response	% viable cells
1	IIIA	Adenocarcinoma	Carboplatin	Unknown (unsufficient tumor cells)	**PD**	No surgery
2	II	Adenocarcinoma	Carboplatin	Negative	**SD**	**60**
3	IIIA	Adenocarcinoma	Cisplatin	Negative	**PR**	**5**
4	II	Adenocarcinoma	Carboplatin	Negative	**SD**	**80**
5	II	Adenocarcinoma	Carboplatin	Positive (5%)	**SD**	**15**
6	IIIA	Adenocarcinoma	Carboplatin	Negative	**PD**	**40**
7	IIIA	Adenocarcinoma	Carboplatin	Negative	**SD**	No surgery
8	IIIA	Adenocarcinoma	Carboplatin	Positive (1%)	**PR**	No surgery
9	IB	Adenocarcinoma	Cisplatin	Negative	**SD**	**80**
10	II	Adenocarcinoma	Carboplatin	Unknown (insufficient tumor cells)	**PD**	**5**
11	II	Adenocarcinoma	Carboplatin	Negative	**SD**	**95**
12	IIIA	Adenocarcinoma	Carboplatin	Negative	SD	No surgery
13	IIIA	Squamous Cell	Carboplatin	Positive	**CR**	**0**
14	IB	Adenocarcinoma	Cisplatin	Negative	SD	90
15	IIIA	Squamous Cell	Carboplatin	Positive (20%)	**PR**	15

Note

Abbreviations: CR, complete response; PD, progressive disease; PR, partial response; SD, stable disease.

Since resectability of the tumor by thoracic surgery was one of the inclusion criteria, pathological response is one of the secondary endpoints for this study. Therefore, the correlation between pathological response and radiological response can be done. The patient with a radiologic CR had also a complete pathological response. Among the three patients who had PR, one patient had a major pathological response, one patient did not have a major pathological response, and one patient could not be assessed pathologically posttreatment because of his ineligibility due to surgery. Among the eight patients with SD, two patients were not assessed because of their ineligibility for surgery and none of the remaining six patients had a major or complete response. Among the three patients with PD, two patients underwent surgery; one patient had a major pathological response, and the other did not have a major pathological response. This correlation is limited due the insufficient data and the ineligibility of patients to surgery from all the groups of radiological responses. Although the pathological response results from this study, considering complete and major pathological response, are limited due to the previously mentioned reasons, it failed to achieve a high overall pathological response rate (27.27%) when compared to the rate achieved in NADIM trial (84.6%).^17^

The RECIST 1.1 measurements for the patient with PD and major pathological response showed an increase of 85% in the size of the target lesion (Figure 1).

Four out of the 15 patients (26.70%) who participated in this study did not proceed to surgery. This rate is high compared to other similar studies. NADIM study reported only five patients out of 46 (10.87%) who did not proceed to surgery after neoadjuvant chemo‐immunotherapy.^17^ All 20 patients who participated in CheckMate‐159 underwent surgical resection after immunotherapy.^16^ About 18.39% of patients did not proceed to surgery in the study^18^ after chemotherapy. The high percentage of patients who did not proceed to surgery after chemoimmunotherapy in our study was due to either progression of the disease (patient 1 in Table 2) or poor lung function tests postchemoimmunotherapy and failure to convert the planned resection from a pneumonectomy to a lobectomy (patients 7, 8, and 12 in Table 2). Other similar studies proceeded with the thoracic surgery extent based on baseline imaging assessment only. This strategy among other factors contributed to the increase in rate of patients proceeding to thoracic surgery (82%).^18^

This study confirms that the preoperative administration of chemotherapy and avelumab is safe with no significant morbidity. There was no indication of increased surgical complications including infections, wound dehiscence, or hospital stay.

Multiple ongoing larger phase III studies are underway testing the efficacy and safety of combination of chemotherapy and immunotherapy and these will help better define the role of this approach in patients with resectable NSCLC.

## CONFLICT OF INTEREST

The authors declare no conflict of interest.

## AUTHORS' CONTRIBUTION

Arafat Tfayli with the help of Majd Al Assaad, Ghina Fakhri, Reem Akel, Hanine Atwi designed the study, coordinated the clinical trial implementation and execution in multiple sites, and wrote the manuscript. Hady Ghanem, Fadi El Karak, Fadi Farhat, and Kamal Al Rabi recruited patients and contributed to the management of the clinical trial in multiple sites. Pierre Sfeir, Pierre Youssef, and Ziad Mansour performed the surgeries for the patients included in the study. Mohamad Haidar and Alain Abi Ghanem assessed the radiological exams of the study patients. Ibrahim Khalifeh, Fouad Boulos, and Ramy Mahfouz contributed to the pathological assessment of the patients. Bassem Youssef and Youssef Zeidan helped in the assessment and management of the patients. Rachelle Bejjany contributed to management of the data and coordination between multiple sites. Fadlo Khuri and Hazem Assi contributed to reviewing and modifying the manuscript.

## Data Availability

Data that support the findings of this study are available on request from the corresponding author, AT The data are not publicly available as it contains confidential information that could compromise the privacy of research participants.
